# The neutralizing antibody response post COVID-19 vaccination in patients with myeloma is highly dependent on the type of anti-myeloma treatment

**DOI:** 10.1038/s41408-021-00530-3

**Published:** 2021-08-02

**Authors:** Evangelos Terpos, Maria Gavriatopoulou, Ioannis Ntanasis-Stathopoulos, Alexandros Briasoulis, Sentiljana Gumeni, Panagiotis Malandrakis, Despina Fotiou, Eleni-Dimitra Papanagnou, Magdalini Migkou, Foteini Theodorakakou, Maria Roussou, Evangelos Eleutherakis-Papaiakovou, Nikolaos Kanellias, Ioannis P. Trougakos, Efstathios Kastritis, Meletios A. Dimopoulos

**Affiliations:** 1 Department of Clinical Therapeutics, School of Medicine, National and Kapodistrian University of Athens, Athens, Greece; 2 Department of Cell Biology and Biophysics, Faculty of Biology, National and Kapodistrian University of Athens, Athens, Greece

**Keywords:** Myeloma, Antibodies

## Abstract

Recent data suggest a suboptimal antibody response to COVID-19 vaccination in patients with hematological malignancies. Neutralizing antibodies (NAbs) against SARS-CoV-2 were evaluated in 276 patients with plasma cell neoplasms after vaccination with either the BNT162b2 or the AZD1222 vaccine, on days 1 (before the first vaccine shot), 22, and 50. Patients with MM (*n* = 213), SMM (*n* = 38), and MGUS (*n* = 25) and 226 healthy controls were enrolled in the study (NCT04743388). Vaccination with either two doses of the BNT162b2 or one dose of the AZD1222 vaccine leads to lower production of NAbs in patients with MM compared with controls both on day 22 and on day 50 (*p* < 0.001 for all comparisons). Furthermore, MM patients showed an inferior NAb response compared with MGUS on day 22 (*p* = 0.009) and on day 50 (*p* = 0.003). Importantly, active treatment with either anti-CD38 monoclonal antibodies (Mabs) or belantamab mafodotin and lymphopenia at the time of vaccination were independent prognostic factors for suboptimal antibody response following vaccination. In conclusion, MM patients have low humoral response following SARS-CoV-2 vaccination, especially under treatment with anti-CD38 or belamaf. This underlines the need for timely vaccination, possibly during a treatment-free period, and for continuous vigilance on infection control measures in non-responders.

## Introduction

The novel coronavirus severe acute respiratory syndrome coronavirus 2 (SARS-CoV-2) has led to a worldwide pandemic and has become a major global health concern. The coronavirus genome encodes four different main structural proteins designated as spike (S), envelope, membrane, and nucleocapsid. The virus penetrates through the viral S protein to the angiotensin-converting enzyme 2 (ACE2) receptors that are mainly presented on oral mucosa epithelial cells and lung alveolar type II cells as well as in other human tissues [1, 2]. COVID-19 is a systemic disease with both short- and long-term manifestations [3, 4]. Most of the patients will present with mild or moderate symptoms, although up to 5–10% will present with severe or life-threatening disease course. The development of effective and safe vaccines, as well as novel therapeutics, became a global priority [5, 6].

Immunocompromised patients with hematological malignancies or solid cancer are more susceptible to infection from SARS-CoV-2 and at higher risk of severe complications and worse outcomes compared with the general population [7, 8]. However, there is significant heterogeneity among different cancer subgroups [9]. Patients with hematological malignancies seem to be more susceptible to SARS-CoV-2 with higher morbidity and mortality when compared to patients with solid organ malignancies [10]. Among hospitalized patients with COVID-19 and hematological cancers, the risk of death has been estimated ~39% [10]. Furthermore, lower seroconversion rates following COVID-19 have been reported among patients with solid and hematological cancer compared with convalescent individuals without cancer [11–14].

Patients with multiple myeloma (MM) are at increased risk of infections due to their immunocompromised state, older age, and comorbidities [15]. COVID-19 causes moderate to severe acute respiratory dysfunction in 77% of MM patients and leads to critical condition in ~8% of them [16]. More than 80% of MM patients who are infected by SARS-CoV-2 require hospital admission [17], while almost 33% of hospitalized MM patients with COVID-19 may die because of the infection [8].

Vaccination against SARS-CoV-2 constitutes an important preventive strategy against COVID-19 in patients with MM, in addition to other precaution measures including mask wearing, social distancing, and modifications in the treatment schedule during the pandemic [18, 19]. However, its efficacy in MM is largely unknown [20–22]. In general, patients with MM may present a decreased antibody response following vaccination [23, 24]. This is attributed to defects in immune effector cells, associated with the B-cell disorder and the use of anti-myeloma regimens [14]. The BNT162b2 mRNA and the AZD1222 viral vector vaccines against SARS-CoV-2 have shown significant efficacy in healthy adults [25, 26]. The first BNT162b2 dose confers some protection among nursing facility members, health workers, and octogenarians [27–29]. However, low antibody responses have been demonstrated among 48 elderly myeloma patients who received the first dose of the BNT162b2 vaccine [30]. In another study including 42 patients with MM, it was demonstrated that they had suboptimal responses after vaccination with the BNT162b2 mRNA vaccine, especially those on treatment with anti-CD38-based regimens [31]. Regarding ChAdOx1 nCoV-19 vaccine (AZD1222), no data on its efficacy among patients with solid and hematological malignancies are currently available.

In this context, we report here the development of neutralizing antibodies (NAbs) against SARS-CoV-2 in patients with plasma cell neoplasms after vaccination with either the mRNA BNT162b2 or viral vector AZD1222 vaccine, up to day 50 post their first vaccine dose, and we evaluate possible correlations with clinical characteristics of patients as well as with treatment data.

## Patients and methods

### Patients and controls

Major inclusion criteria for the participation of patients in this study included: (i) age above 18 years; (ii) presence of monoclonal gammopathy of undetermined significance (MGUS), smoldering myeloma (SMM) who have never received any kind of anti-myeloma therapy or active MM according to International Myeloma Working Group criteria [32], irrespective of the treatment given or the line of therapy; and (iii) eligibility for vaccination, according to International Myeloma Society recommendations [33]. Volunteers of similar age and gender, who served as controls, were also included in this analysis.

Major exclusion criteria for both myeloma patients and controls included the presence of: (i) autoimmune disorder or other active malignant disease; (ii) HIV or active hepatitis B and C infection, (iii) end-stage renal disease, and (iv) prior diagnosis of COVID-19. Relevant data was extracted from the medical records and included: demographics, complete blood count, serum immunoglobulin (Ig) levels, disease status, and type of treatment. Dexamethasone administration was held 2 weeks before until 1 week after each vaccine shot.

All participants have been enrolled in a large prospective study (NCT04743388) evaluating the kinetics of anti-SARS-CoV-2 antibodies after COVID-19 vaccination in healthy subjects and in patients with hematological malignancies or solid tumors. The study was approved by the Institutional Ethics Committee of General Hospital Alexandra, Athens, Greece in accordance with the Declaration of Helsinki and the International Conference on Harmonization for Good Clinical Practice. All patients and controls provided written informed consent prior enrollment in the study.

### NAbs measurement

After vein puncture, the serum of both patients and controls was collected on day 1 (D1; before the first BNT162b2 or AZD1222 dose), on day 22 (D22; before the second dose of the BNT162b2 or 3 weeks post the first AZD1222 dose) and on day 50 (D50; 4 weeks post second dose of the BNT162b2 or 7 weeks post the first AZD1222 dose).

Serum was separated within 4 h from blood collection and stored at −80 °C until the day of measurement. NAbs against SARS-CoV-2 were measured using FDA approved methodology (ELISA, cPass™ SARS-CoV-2 NAbs Detection Kit; GenScript, Piscataway, NJ, USA) [34] on the abovementioned timepoints. A NAb titer of at least 30% is considered as positive, whereas a NAb titer of at least 50% has been associated with clinically relevant viral inhibition [35]. According to FDA, high NAb titer for this specific method is considered any value above or equal to 68%. Samples of the same patient or control were measured on the same ELISA plate.

### Statistical analysis

All statistical analyses were performed with STATA (version 17.0, College Station, Texas). All variables were tested for normal data distribution. Normally distributed data were expressed as means ± standard deviation (SD). Non-normally distributed data were presented as the median with the interquartile range (IQR). For categorical variables, the *χ*^2^ or Fisher exact test were used to compare the distributions for the two randomized groups. Non-paired Student’s *t* tests were used for between-treatment comparisons of continuous variables. Post hoc mixed-model repeated measures analysis was used to evaluate the neutralizing antibodies over time with cases and controls as main effects and neutralizing antibodies as dependent variables. Mixed models were performed using direct likelihood estimation with fixed effects of groups, time of antibodies and interaction of groups (cases, controls) by timing of antibody measurement. An unstructured covariance matrix was used to model within-patient error. All significance tests were two tailed and conducted at the 5% significance level.

## Results

### Baseline characteristics of patients and controls

Study population included 276 patients with plasma cell neoplasms (213 symptomatic MM, 38 SMM, 25 MGUS) (151 males/125 females; median age: 74 years, IQR: 62–80 years) and 226 controls matched for age and gender who were vaccinated during the same period, at the same vaccination center (Alexandra Hospital, Athens, Greece).

Two hundred and fifteen patients (77.9%) were vaccinated with the BNT162b2 and 61 (22.1%) with the AZD1222 vaccine. The relative proportion in the control group was similar with the patient group; 171 (75.66%) were vaccinated with the BNT162b2 and 55 (24.34%) with the AZD1222 vaccine (*p* = 0.56). The median BMI between the two groups was similar [26 (IQR 24–29) for the patient group versus 27 (IQR 23–30) for the control group, *p* = 0.8]. Forty-two patients (15.22%) had lymphopenia (<1000 lymphocytes/mm^3^), of which 40 out of 42 were symptomatic MM patients. At the time of vaccination 34 (12.32%) patients with MM were not receiving active treatment.

The baseline characteristics of the enrolled patients are depicted on Table 1.

**Table 1:** Baseline characteristics of patients with MM/SMM/MGUS.

Number of patients	*N* = 276
	MM *n* = 213 (77.2%)
	SMM *n* = 38 (13.7%)
	MGUS *n* = 25 (9.1%)
Gender	Male *n* = 151 (54.7%)
	Female *n* = 125 (45.3%)
Age in years, median (range)	74 (IQR: 62–80)
BMI, median (range)	26 (IQR: 24–29)
Comorbidities	Pulmonary disease: *n* = 18 (6.6%)
	Diabetes mellitus: *n* = 39 (14.3%)
	Autoimmune disease: *n* = 6 (2.3%)
	Cardiovascular disease: *n* = 149 (54.1%)
Total lymphocyte count, median (range), cells/mm^3^	1400 (110–2000)
Lymphopenia (<1000 cells/mm^3^)	*n* = 42 (15.2%) (*n* = 40 MM)
Immunoglobulins, median (range), mg/dl	IgG: 939 (529–1300)
	IgA: 99 (37–217)
	IgM: 27.5 (19–55)
Immunoparesis 1 IgG < 700 mg/dl	*n* = 85 (81 MM, 3 MGUS, 1 SMM) (30.8%)
Immunoparesis 2 IgA < 70 mg/dl	*n* = 112 (98 MM, 2 MGUS, 12 SMM) (40.6%)
Immunoparesis 3 IgM < 40 mg/dl	*n* = 32 (32 MM) (11.5%)
Ig type	
IgA	*n* = 30 (10.8%)
IgG	*n* = 171 (62%)
IgM	*n* = 6 (2.1%)
KLC	*n* = 59 (21.5%)
LLC	*n* = 10 (3.6%)
MM	
ISS-1	*n* = 83 (39.1%)
ISS-2	*n* = 74(34.9%)
ISS-3	*n* = 55 (26%)
RISS-1^a^	*n* = 41 (27.5%)
RISS-2	*n* = 88 (59%)
RISS-3	*n* = 20 (13.5%)
MM Treatment line, median (range)	2 (IQR: 1–3)
MM	
MM off treatment	*n* = 34 (16%)
Belantamab monotherapy	*n* = 3 (1.4%)
Belantamab combinations	*n* = 8 (3.8%)
Daratumumab monotherapy	*n* = 9 (4.2%)
Daratumumab+PI	*n* = 14 (6.6%)
Anti-CD38+IMID	*n* = 29 (13.6%)
Other anti-CD38 combinations	*n* = 3 (1.4%)
Other IMID-based combinations	*n* = 26 (12.2%)
Other PI-based combinations	*n* = 16 (7.5%)
PI and IMID-based combinations	*n* = 37 (17.4%)
Lenalidomide maintenance	*n* = 33 (15.5%)
Cyclophosphamide-Dexamethasone	*n* = 1 (0.4%)
Vaccination with BNT162b2	*n* = 215 (77.8%)
Vaccination with AZD1222	*n* = 61 (22.2%)

^a^Available data for RISS only for 149 patients.

*MM* multiple myeloma, *SMM* smoldering multiple myeloma, *MGUS* monoclonal gammopathy of undetermined significance, *BMI* body mass index, *ISS* International Staging System, *R-ISS* Revised International Staging System, *IgG* immunoglobulin G, *IgA* immunoglobulin A, *IgM* immunoglobulin M, *KLC* kappa light chain, *λLC* lamda light chain, *PI* proteasome inhibitor; *IMiD* immunomodulatory drug.

### Humoral response in patients and controls

**Fig. 1:**
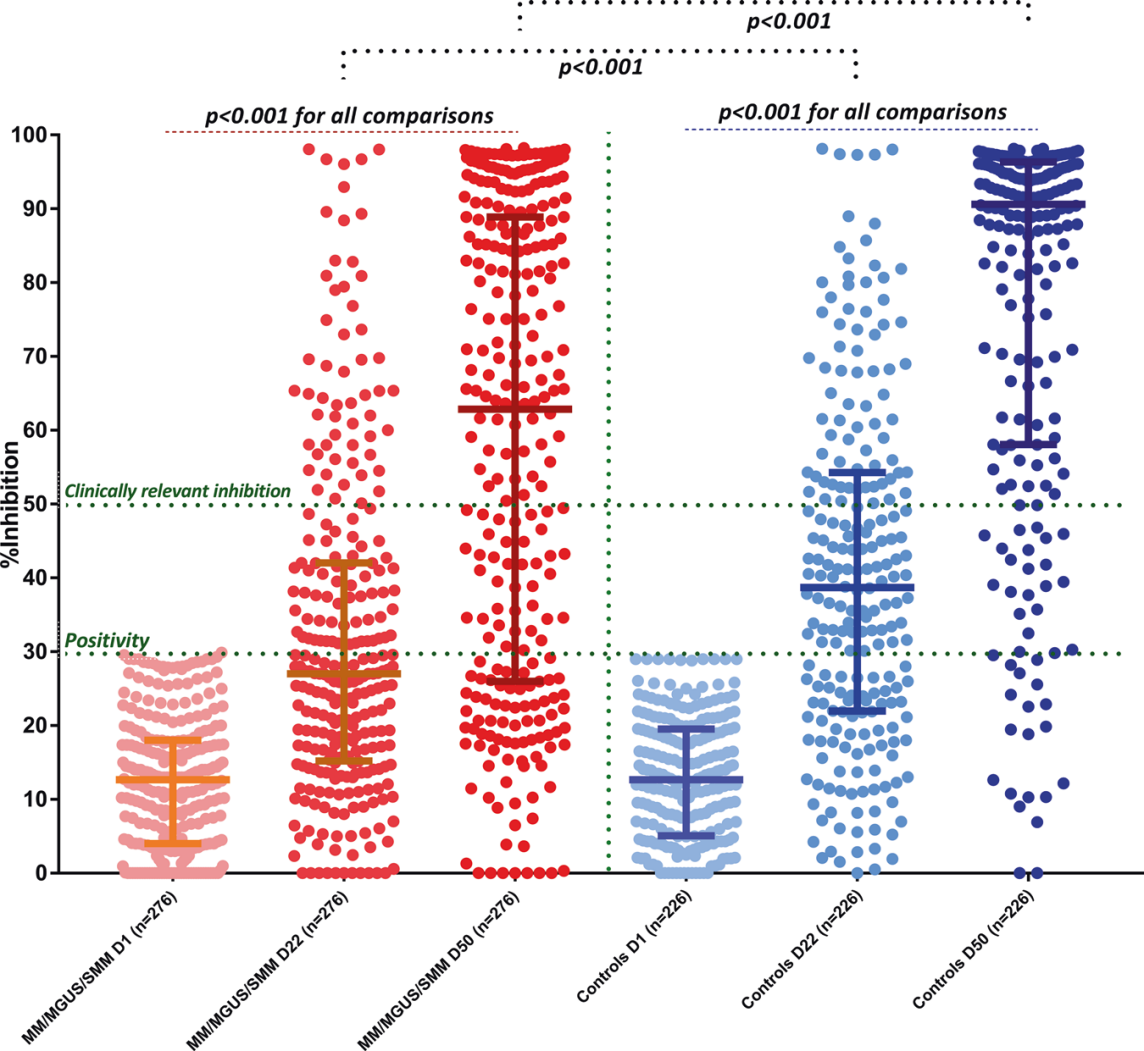
Kinetics of NAbs in MM/SMM/MGUS patients compared with age- and sex-matched controls after vaccination with 2 doses of the BNT162b2 or 1 dose of the AZD1222 vaccine. Patients had significantly lower NAbs titers on day 22 and on day 50 compared with controls.

On D1 there was no difference regarding the NAb titers between patients and controls (*p* = 0.7). After the first dose of the vaccine, on D22, the patient group had lower NAb titers compared with controls: the median NAb inhibition titer was 27% (IQR: 15.3–42%) for patients versus 38.7% (IQR: 22–54.3%) for controls; *p* < 0.001 (Fig. 1). More, specifically, 117 (42.4%) patients versus 145 (64.2%) controls developed NAb titers ≥30% on D22 (*p* < 0.001). The respective number of patients and controls who developed NAb titers ≥50% (clinically relevant viral inhibition) was 55 (19.9%) and 73 (32.3%), respectively (*p* = 0.002). There was no difference between patients who received the BNT162b2 or the AZD1222 vaccine in terms of the values of NAbs titers on D22, or the number of patients who developed NAbs titers ≥30% or ≥50%.

On D50, 4 weeks after the second dose of the BNT162b2 vaccine or 7 weeks after the first dose of AZD1222, the patient group continued to have lower NAb titers compared with controls: the median NAb inhibition titer was 62.8% (IQR: 26–88.9%) for patients versus 90% (IQR: 58–96.4%) for controls; *p* < 0.001 (Fig. 1). More, specifically, 196 (71%) patients versus 204 (90.3%) controls developed NAb titers ≥30% on D50 (*p* < 0.001). The respective number of patients and controls who developed NAb titers ≥50% was 158 (57.3%) and 183 (81%) (*p* < 0.001). Out of these 158 patients, 114 had symptomatic MM, 23 asymptomatic MM, and 21 MGUS and among patients with different plasma cell dyscrasias, 53.5% of symptomatic myeloma patients, 84% of MGUS and 60.5% of those with SMM achieved clinically relevant antibody response (*p* = 0.013). MGUS patients had no differences from controls regarding the development of Nabs titers of ≥30% or ≥50% on D50.

**Fig. 2:**
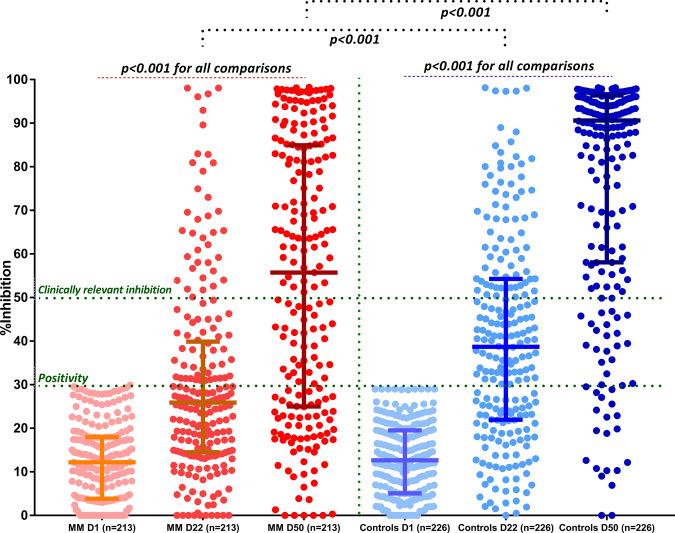
Kinetics of NAbs in patients with symptomatic MM compared with age- and sex-matched controls after vaccination with 2 doses of the BNT162b2 or 1 dose of the AZD1222 vaccine. Patients with MM had significantly lower NAbs titers on day 22 and on day 50 compared with controls.

**Fig. 3:**
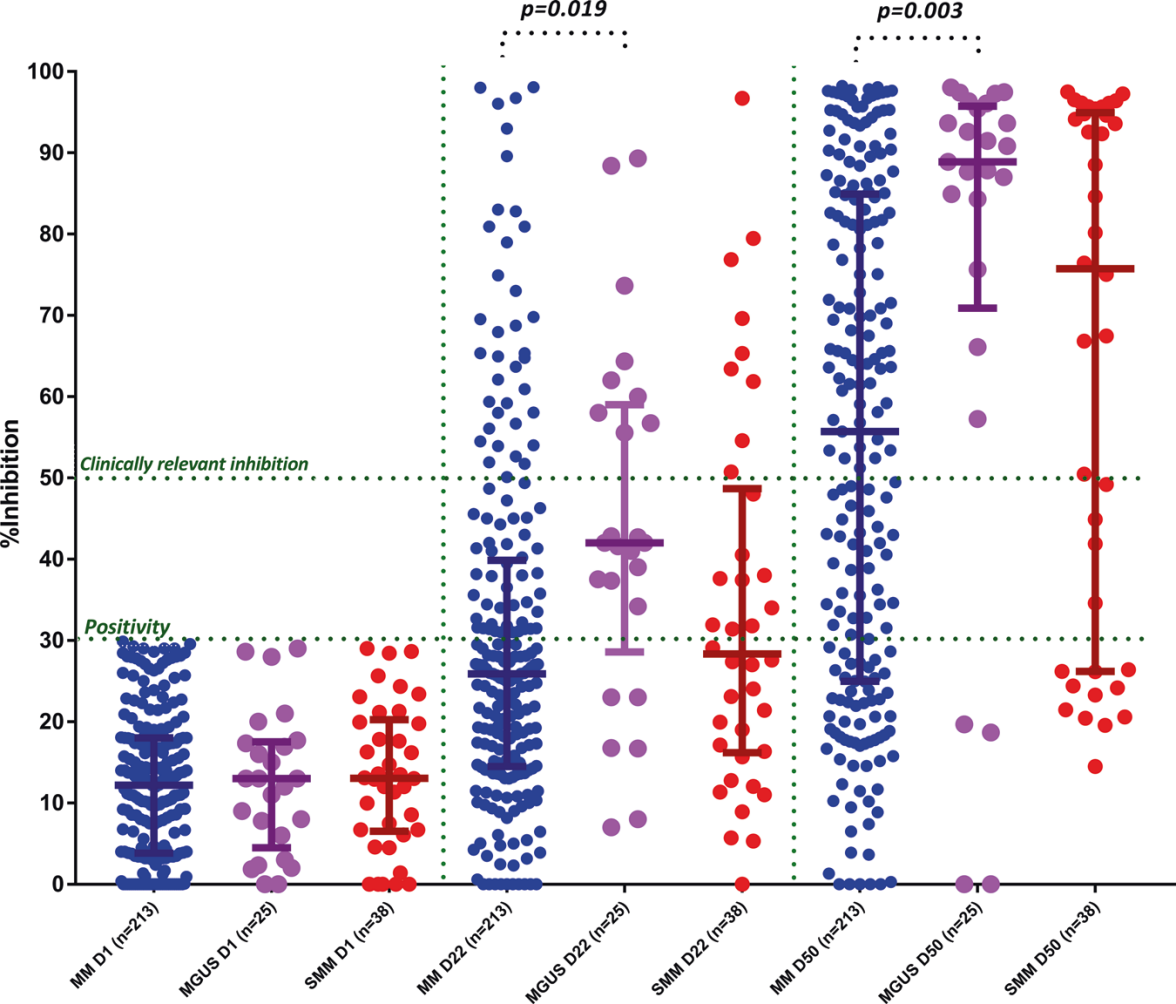
Kinetics of NAbs in MM compared with SMM and MGUS after vaccination with 2 doses of the BNT162b2 or 1 dose of the AZD1222 vaccine. A statistically significant difference was identified both on day 22 and day 50 between the MM and MGUS group.

When we compared only the symptomatic MM patients with the control group, the median NAb inhibition titer was inferior among MM patients compared with controls both on day 22 (25.9%, IQR: 14.6–39.5%, versus 55.7%, IQR: 25–84.8%, *p* < 0.001, respectively) and on day 50 (38.7%, IQR: 22–54.3% versus 90.6%, IQR: 58.1–96.4%, *p* < 0.001, respectively) (Fig. 2). When we compared MM patients with SMM and MGUS on day 22 and day 50, there was no significant difference regarding the NAb production between the group of MM and SMM at all timepoints. On the contrary, there were significant differences in NAb responses between MM and MGUS at both timepoints (mean difference 14%, *p* = 0.009, on day 22 and 21.59%, *p* = 0.003, on day 50, respectively) (Fig. 3).

### Predictive factors for NAbs production

Among the 114 MM patients with clinically relevant response on day 50 (i.e with NAbs titers ≥50%), 23 (20.2%) were in remission, without receiving any therapy (in complete or partial remission) and 91 were on treatment (Table 2). All patients in remission had normal uninvolved immunoglobulins levels post treatment completion and had not received treatment for more than 6 months. Among symptomatic MM patients with low response rates (<30%) on day 50 (*n* = 65), only 2 were off treatment, while 63 were on active treatment (Table 2).

**Table 2:** Type of treatment for NAbs responders and non-responders on day 50.

Day 50, *n* (%)	NAbs >50% (*n* = 114) (%)	NAbs <30% (*n* = 65) (%)
Off treatment	23 (20.2)	2 (3.1)
Belantamab mafodotin monotherapy	0	2 (3.1)
Belantamab mafodotin combinations	2 (1.7)	5 (7.7)
Daratumumab monotherapy	2 (1.7)	6 (9.2)
Daratumumab + PI combinations	5 (4.4)	9 (13.9)
Anti-CD38 antibodies + IMID combinations	14 (12.3)	9 (13.9)
Other anti-CD38 combinations	2 (1.8)	1 (1.5)
IMID-based regimens	11 (9.6)	9 (13.9)
PI-based regimens	6 (5.2)	7 (10.7)
IMID and PI combinations	25 (21.9)	10 (15.3)
Lenalidomide maintenance	23 (20.3)	5 (7.7)
Cyclophosphamide	1 (0.9)	–

*PI* proteasome inhibitor, *IMID* immunomodulatory drug.

On day 22, there was no significant difference in NAbs production among the different myeloma treatment groups. On the other hand, on day 50, patients who did not receive any treatment achieved significantly higher NAbs responses (mean ± SD: 66% ± 25.4%) compared with the patients receiving belantamab mafodotin-based combinations (mean ± SD: 28.2% ± 20.1%, *p* = 0.002, ANOVA pair group comparison) or anti-CD38-based combinations (mean ± SD: 45.4% ± 29.4%, *p* = 0.013, ANOVA pair group comparison). Similar differences were identified for the lenalidomide maintenance group compared with the belantamab and anti-CD38 groups (*p* = 0.003 and *p* = 0.015, respectively, ANOVA pair group comparisons).

**Fig. 4:**
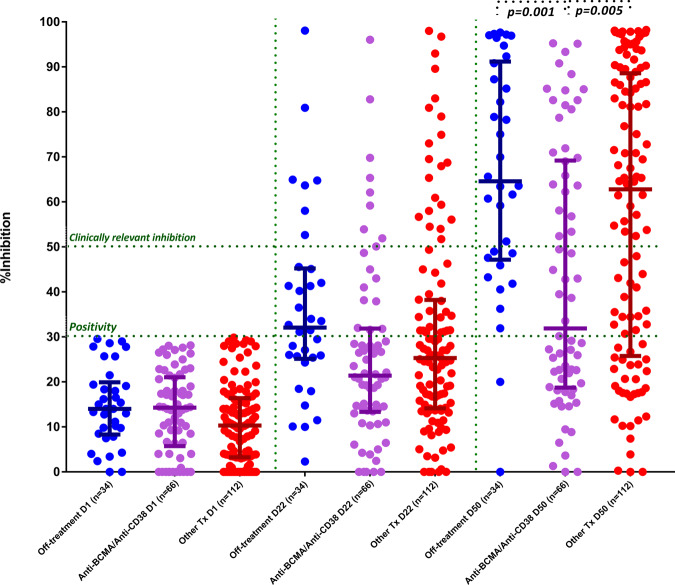
Kinetics of NAbs in symptomatic MM patients on treatment with Belantamab mafodotin or anti-CD38-based combinations compared with MM patients off treatment and MM patients on treatment with other therapeutic regimens. Patients on Belantamab mafodotin or anti-CD38 combinations had significantly lower NAbs on day 50.

When we compared the group of patients on treatment with belantamab mafodotin or anti-CD38 monoclonal antibodies with those receiving other treatment regimens and those who did not receive any treatment, there was a decreased median NAb titer on day 50 for the belantamab/anti-CD38 group compared with the other two groups. More specifically, the median NAb titer was 31.9% (IQR: 18.9–69) for the belantamab/anti-CD38 group compared with 62.8% (IQR: 26–88.3%) for the other treatment group (*p* = 0.005) and 64.6% (IQR: 47.6–90.8%) for the off-treatment group (*p* = 0.001) (Fig. 4). No other significant findings were noted among treatment groups on day 50.

Furthermore, we sought to investigate predictive factors for NAb titers less than 50% among patients with symptomatic MM on day 50 (Table 3). Age, BMI, ISS, and RISS were not proven as statistically significant in the univariate analysis. On the contrary, treatment type and, more specifically, the combinations based on belantamab mafodotin and anti-CD38 monoclonal antibodies were significant predictive factors for lower antibody response (OR: 9.4, 95% CI: 1.7–51.1, *p* = 0.009, and OR 2.9, 95% CI: 1.2–7.1, *p* = 0.002, respectively). The presence of lymphopenia at the time of vaccination was also a significant predictive factor for decreased humoral response (OR: 3.5, 95% CI: 1.8–6.7, *p* = 0.019), as well as the low levels of IgA (OR: 2.9, 95% CI: 1.8–4.4, *p* = 0.024). Female gender was predictive for superior NAb production on day 50 compared with males (OR: 0.6, 95% CI: 0.4–0.9). Importantly, the multivariate analysis showed that treatment with combinations based on belantamab mafodotin (OR 7.6, 95% CI: 1.4–42.4 *p* = 0.021) and lymphopenia (OR: 2.1, 95% CI: 1.0–4.5, *p* = 0.048) were the only significant predictive factors for NAb response on day 50. There was also a trend towards increased risk for poor NAb response following COVID-19 vaccination with anti-CD38-based regimens (OR: 2.4, 95% CI: 2.9–6.2, *p* = 0.07) (Table 3). At this point, we need to stress that almost 85% of patients under daratumumab-based regimens were at the monthly administration schedule of daratumumab.

**Table 3:** Univariate and multivariate analysis for NAbs titers <50% among patients with symptomatic MM on day 50.

Variable	Univariate analysis OR, 95% CI	Multivariate OR, 95% CI
Age	0.99, 0.98–1.01	–
Gender	–	–
Males (reference)		
Females	*0.6, 0.4–0.9*	–
BMI	1.03, 0.98, 1.09	–
Disease type (vs. controls)		Omitted due to collinearity
Multiple myeloma	*3.7, 2.4–5.7*	–
MGUS	0.8, 0.3–2.5	–
Smoldering multiple myeloma	*2.8, 1.3–5.8*	–
ISS		–
Stage I (reference)		
Stage II	0.76, 0.4–1.4	–
Stage III	1.5, 0.8–3	–
RISS		
Stage I (reference)	–	–
Stage II	1.7, 0.8–3.5	–
Stage III	1.7, 0.6–5.1	–
Myeloma type		
IgG (reference)		
IgA	1.07, 0.5–2.3	–
IgM	0.75, 0.1–4.7	–
KLC	1, 0.4–2.5	–
LLC	1.9, 0.7–3.5	–
Lymphopenia (<1000 cells/cm3)	*3.5, 1.8*–*6.7*	*2.1, 1–4.5*
Immunoparesis		
IgA<70 mg/dl	*2.9, 1.8–4.4*	–
IgM<40 mg/dl	1	–
IgG<700 mg/dl	1.7, 0.4-3.1	–
Treatment type		
Off-treatment (reference)		
Anti-BCMA-based regimens	*9.4, 1.7*–*51.1*	*7.6, 1.4*–*42.4*
Anti-CD38-based regimens	*2.9, 1.2–7.1*	2.4, 2.9–6.2*
PI/IMID-based combinations	1.8, 0.8–4.3	–
Lenalidomide maintenance	0.9, 0.3–2.6	–

*MM* multiple m yeloma, *BMI* body mass index, *ISS* International Staging System, *R-ISS* Revised International Staging System, *IgG* immunoglobulin G, *IgA* immunoglobulin A, *IgM* immunoglobulin M, *PI* proteasome inhibitor, *IMiD* immunomodulatory drug, *OR* odds ratio, *CI* confidence interval.

*p = 0.07.

Italics denote statistical significance.

We subsequently examined if there was any effect of the line of therapy (two versus more than two lines of therapy or three versus more than three lines) and there was no influence of the line of therapy on the development of NAbs in all studied timepoints.

### Adverse events

Seventy-one (33%) and 68 patients (31.6%) reported mild reactions after the first and second dose of the BNT162b2 vaccine, respectively. Twenty (32.8%) patients vaccinated with the first dose of AZD1222 also presented with local reactions. These reactions included mainly pain at the site of the injection, erythema, and/or swelling. The rate of this adverse event between the first and second dose of the BNT162b2 was not statistically significant (*p* = 0.7). In all, 13% (*n* = 28) and 21% (*n* = 45) of the patients vaccinated with the BNT162b2 vaccine reported systemic adverse reactions but all were categorized as mild (grade 1 or 2). The systemic adverse events included fatigue, fever, lymphadenopathy, muscle pain, arthargias, and headache. The presence of the adverse events was independent of the active treatment or disease status.

## Discussion

Our data indicate that vaccination with either the BNT162b2 mRNA or the AZD1222 viral vector vaccine leads to a less intense humoral response, as reflected by a lower production of NAbs, against SARS-CoV-2 among patients with MM/SMM compared with healthy controls of similar age and gender and without malignant disease. However, patients with MGUS did not have significant differences compared with healthy controls. Our findings were independent of the vaccine type on D22, where we could perform a fair comparison (3 weeks after the first vaccine dose; on D50 patients had received two doses of BNT162b2 bur only one dose of AZD1222). Active treatment with regimens including belantamab mafodotin or anti-CD38 monoclonal antibodies that deplete B-cells, as well as lymphopenia, were negative prognostic factors at the multivariate analysis, as they were correlated with lower antibody response rates. To our knowledge, this is the first report to demonstrate the kinetics of NAbs-mediated humoral response in patients with MM after 2 doses of vaccination with the BNT162b2 vaccine and the first dose of AZD1222.

The underlying causes for low humoral response to vaccination in patients with plasma cell dyscrasias are multifactorial and it seems that both disease-related immune dysregulation and therapy related immunosuppression are involved [23]. Interestingly, both MM and SMM patients showed a suboptimal humoral immune response following vaccination, suggesting that the disease itself plays a crucial role in immunosuppression of these patients. Myeloma cells suppress normal B-cell expansion and immunoglobulin production. MM is a de novo immunological disease with impaired function of immune cells in the marrow microenvironment characterized by dysfunction of effector cells, loss of antigen presentation and expansion of immunosuppressive cells [36]. Memory B-cell and T-cell responses might be significantly compromised in patients with MM [37]. Furthermore, anti-myeloma B-cell depleting therapies may impair immune response to vaccines, whereas both myeloma microenvironment and anti-myeloma treatment may impair T-cell function as well [23].

Patients with MM often present suboptimal seroconversion rates after a single-dose vaccine against bacteria and viruses and, therefore, booster doses are needed to assure adequate protection, such as the case with the seasonal flu vaccine [23]. A single dose of inactivated influenza vaccine in patients with MM result in a low seroconversion rate reaching 20–25% [38]. Interestingly, studies that evaluated the role of a booster influenza dose demonstrated increased immunity reaching at 70% [39, 40]. Active disease and active treatment were associated with lower response rates.

These results are in accordance with our study demonstrating the low humoral response with the COVID-19 vaccines. Patients receiving anti-myeloma treatment without any anti-CD38 regimens were more likely to respond to vaccination. On the other hand, daratumumab- or isatuximab-based and anti-BCMA-based regimens were significantly associated with decreased NAbs responses, probably due to a direct effect on the immune system. Anti-CD38 monoclonal antibodies and anti-BCMA compounds directly reduce immunogenicity by depleting antibody-producing B-cells [41–44]. However, a recent study showed that antibody production against Hemophilus Influenzae B, seasonal influenza, and Streptococcus pneumoniae was not compromised when the vaccination was performed at least 2 months post the last dose of daratumumab [45]. On the contrary, IMiDs promote the activation of the immune system especially via T and NK cells enhancement, cytokine increase, decreased Treg activity and enhancement of dendritic cells and antibody-dependent cell-mediated cytotoxicity, which is also supported by our results regarding COVID-19 vaccination [46, 47]. In the light of our findings, it may be eloquently supported that a booster dose may be needed to enhance humoral response, especially for patients with MM on treatment with B-cell depleting therapies. If possible, a delay in treatment initiation with such anti-myeloma drugs might be also considered on a tailored basis until the vaccination is completed. Further studies including higher patient numbers may be necessary to determine the optimal dosing, dosing intervals, and number of boosting doses in these patients, as well as treatment-specific intervals before vaccination.

Furthermore, hypoglobulinemia has been associated with inferior antibody responses among patients with other hematological malignancies and COVID-19 [14]. In our study, it has been confirmed that patients with increased humoral responses did not experience immunoparesis. Furthermore, it seems that patients who completed their treatment and remained on response at the time of vaccination were more likely to produce NAbs and this is probably related to a reconstitution of humoral immunity.

Interestingly, it has been previously shown that the production of NAb titers at a level of ≥50% on D22 after the first BNT162b2 dose was low even among healthy individuals aged 65–85 years [35]. However, higher antibody titers after a single dose of mRNA-based vaccine against SARS-CoV-2 have been detected in individuals who have recovered from COVID-19 [48]. Since our results denote that patients with plasma cell dyscrasias have blunted antibody responses, they also suggest that the second timely vaccine dose is necessary for this subpopulation with a malignant hematological disease that deregulates the immune homeostasis. In accordance to a previous study on healthy individuals [49], our results advocate for an earlier administration of the second dose of the AZD1222 especially for patients with MM. It is worth mentioning that our study included only one dose of AZD1222, and, thus, firm conclusions cannot be drawn regarding the humoral response after the full vaccination schedule with this vaccine type. It should be also highlighted that patients with MM and SMM presented with a similar humoral response profile, while patients with MGUS achieved superior antibody responses similar to healthy individuals. Further studies will elucidate if this can be a discriminatory factor regarding the need for booster doses.

One of the main strengths of our study is the evaluation of NAbs, which have been shown to have an important predictive value of immune protection from symptomatic COVID-19 in a recent study [50]. Therefore, NAb levels can be considered valuable surrogates of vaccine efficacy. The main limitations of our study include the relevant limited number of patients in the subgroup analyses, the absence of data on T-cell induced immune responses following vaccination against SARS-CoV-2 and the short follow up period that was not enough to determine the role of vaccination against clinically important COVID-19 infection in patients with plasma cell dyscrasias. In general, the vaccine protection against severe COVID-19 and reinfection by SARS-CoV-2 variants for patients with hematological cancer has not been elucidated yet.

In conclusion, patients with myeloma have an inferior humoral response against SARS-CoV-2 after COVID-19 vaccination compared with healthy individuals. Although humoral immunity seems to be deregulated, mucosal surface antibodies, such as IgA, and protective T-cell responses, might particularly important in the protection following natural SARS-CoV-2 infection or vaccination [51, 52]. Further studies on the kinetics of immune subpopulations following COVID-19 vaccination will elucidate the underlying immune landscape and determine the potential need for additional booster doses or protective administration of antibodies against SARS-CoV-2 in patients with plasma cell dyscrasias and no/poor response after full vaccination. For these patients prolonged self-protection measures, such as mask wearing and social distancing, are necessary. Finally, the effect of the long-term safety of these vaccines seems reassuring, however close monitoring is required especially in patients with plasma cell dyscrasias, considering the increased risk for concurrent or synergistic adverse events.
